# ARGprofiler—a pipeline for large-scale analysis of antimicrobial resistance genes and their flanking regions in metagenomic datasets

**DOI:** 10.1093/bioinformatics/btae086

**Published:** 2024-02-20

**Authors:** Hannah-Marie Martiny, Nikiforos Pyrounakis, Thomas N Petersen, Oksana Lukjančenko, Frank M Aarestrup, Philip T L C Clausen, Patrick Munk

**Affiliations:** Research Group for Genomic Epidemiology, Technical University of Denmark, Henrik Danms Allé, Bygning 204, Kongens Lyngby 2800, Denmark; Research Group for Genomic Epidemiology, Technical University of Denmark, Henrik Danms Allé, Bygning 204, Kongens Lyngby 2800, Denmark; Research Group for Genomic Epidemiology, Technical University of Denmark, Henrik Danms Allé, Bygning 204, Kongens Lyngby 2800, Denmark; Research Group for Genomic Epidemiology, Technical University of Denmark, Henrik Danms Allé, Bygning 204, Kongens Lyngby 2800, Denmark; Research Group for Genomic Epidemiology, Technical University of Denmark, Henrik Danms Allé, Bygning 204, Kongens Lyngby 2800, Denmark; Research Group for Genomic Epidemiology, Technical University of Denmark, Henrik Danms Allé, Bygning 204, Kongens Lyngby 2800, Denmark; Research Group for Genomic Epidemiology, Technical University of Denmark, Henrik Danms Allé, Bygning 204, Kongens Lyngby 2800, Denmark

## Abstract

**Motivation:**

Analyzing metagenomic data can be highly valuable for understanding the function and distribution of antimicrobial resistance genes (ARGs). However, there is a need for standardized and reproducible workflows to ensure the comparability of studies, as the current options involve various tools and reference databases, each designed with a specific purpose in mind.

**Results:**

In this work, we have created the workflow ARGprofiler to process large amounts of raw sequencing reads for studying the composition, distribution, and function of ARGs. ARGprofiler tackles the challenge of deciding which reference database to use by providing the PanRes database of 14 078 unique ARGs that combines several existing collections into one. Our pipeline is designed to not only produce abundance tables of genes and microbes but also to reconstruct the flanking regions of ARGs with ARGextender. ARGextender is a bioinformatic approach combining KMA and SPAdes to recruit reads for a targeted *de novo* assembly. While our aim is on ARGs, the pipeline also creates Mash sketches for fast searching and comparisons of sequencing runs.

**Availability and implementation:**

The ARGprofiler pipeline is a Snakemake workflow that supports the reuse of metagenomic sequencing data and is easily installable and maintained at https://github.com/genomicepidemiology/ARGprofiler.

## Introduction

Investigating the resistome of metagenomic datasets, including the abundances of the different antimicrobial resistance genes (ARGs) and the gene synteny (gene flanking regions), has become a major research area in recent years (Holmes *et al.* 2016, Bengtsson-Palme *et al.* 2018, Hendriksen *et al.* 2019, Anthony *et al.* 2021, Zhang *et al.* 2021, Martiny *et al.* 2022b, Munk *et al.* 2022). In many cases, research investigation, especially on a large scale, has been limited to research groups that are technologically and financially able to combine large-scale data generation with advanced bioinformatic and modeling expertise. However, because of the good data-sharing practices of next-generation sequencing efforts, there are today a large number of sequencing datasets available in public repositories. We have recently provided a curated dataset of acquired ARG abundance estimates in more than 214 000 publicly available metagenomic datasets (Martiny *et al.* 2022a).

Processing these datasets in a uniform approach calls for optimized, standardized methods to support the broader scientific community in utilizing these datasets. The practice of sharing bioinformatic workflows, or pipelines, has not historically been part of the academic publishing process. However, with the growing volumes of biological sequencing data available, researchers have begun to publish their workflows. Recent examples include pangolin for tracing SARS-CoV2 lineages (O’Toole *et al.* 2021), RASflow for RNA sequencing data (Zhang and Jonassen 2020), and ATLAS for metagenomic sequencing data (Kieser *et al.* 2020).

**Figure 1.**
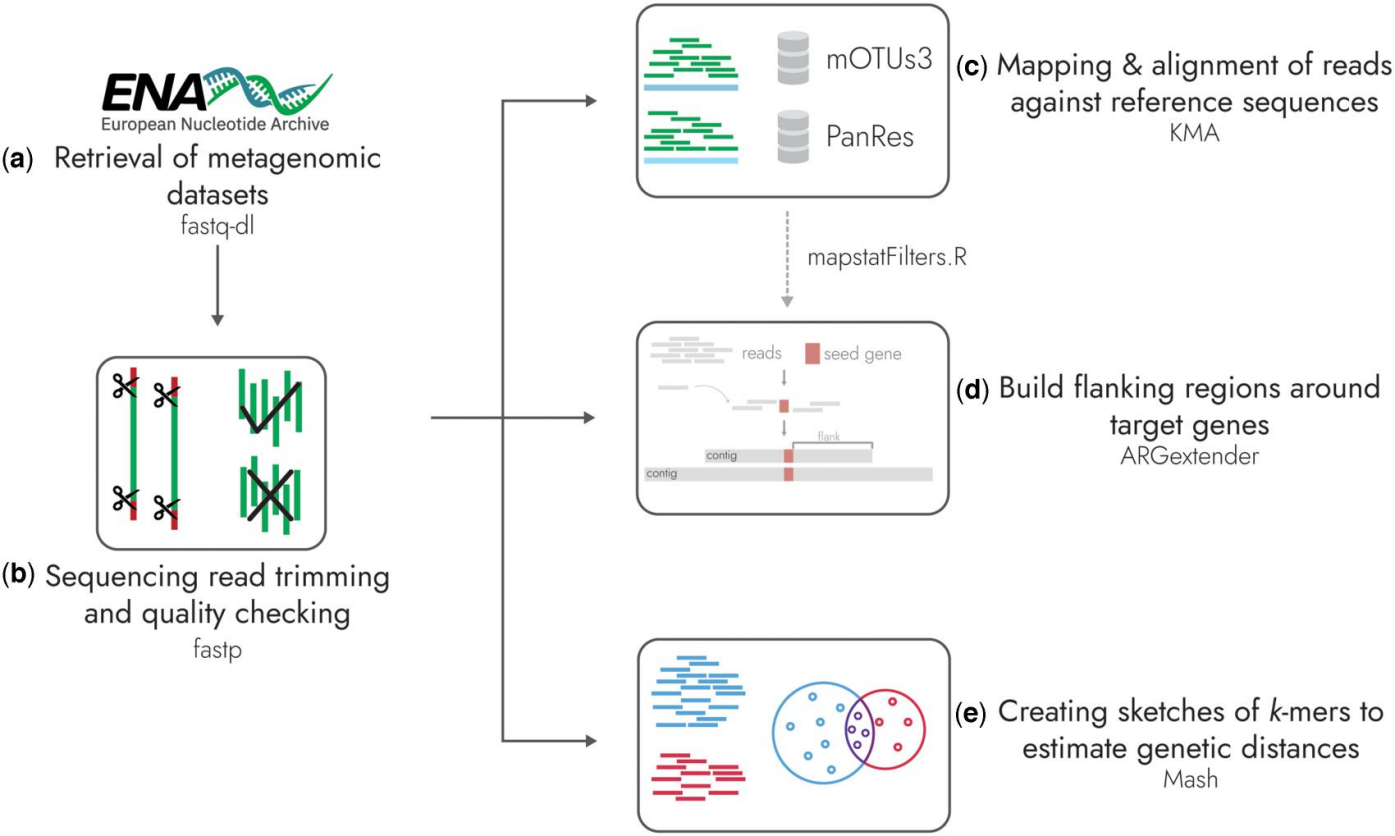
The ARGprofiler pipeline. This schematic illustrates the components of the pipeline: (a) Download of sequencing reads from ENA, (b) preprocessing of retrieved reads with fastp, (c) procedure of aligning reads against chosen reference sequence database(s) with KMA, (d) assembly of targeted flanking regions of antimicrobial resistance genes with ARGextender that passes requirements in a filtering step, and (e) sketching sequencing reads with Mash.

Here, we present ARGprofiler, a newly developed pipeline designed to analyze read dissimilarities, abundances, and genomic flanking regions of ARGs in metagenomic sequencing data (Fig. 1). ARGprofiler has been adapted to work for short-read sequencing reads, where we have carefully evaluated each step in our metagenomic workflow. ARGprofiler includes the PanRes database, a combined collection of current ARG databases, and ARGextender, an assembly tool for producing targeted *de novo* assemblies. The pipeline is an easily usable and scalable workflow implemented in Snakemake (Köster and Rahmann 2012, Mölder *et al.* 2021), which allows any user to process sequencing data to perform epidemiological analyses of ARGs globally. ARGprofiler is another step toward enabling the reuse of metagenomic sequences, and while we have targeted antimicrobial resistance (AMR), the pipeline can be repurposed for other tasks.

## Methods

### Implementation of ARGprofiler

ARGprofiler is a Snakemake workflow (Köster and Rahmann 2012) that is organized into five different parts: (i) download of metagenomic datasets, (b) trimming and quality check of sequencing reads, (iii) mapping and alignment of reads against reference sequences, including ARGs and bacteria, (iv) building of flanking regions around genes of interest, and (v) creation of Mash sketches (Fig. 1). Mapping to bacteria is done to compare the ARG content to the bacterial composition, and Mash sketches to allow for searching for samples with similar or different compositions.

ARGprofiler can handle both short single- and paired-end reads and combines existing and newly established tools and reference databases to produce a single comprehensive analysis pipeline.

### Retrieval of metagenomic datasets

To manage the download of sequencing read data from ENA, ARGprofiler utilizes fastq-dl 2.0.4 (https://github.com/rpetit3/fastq-dl) to retrieve and download the reads by matching the given run_accessions in a JSON input file provided by the user. ARGprofiler is also capable of handling reads stored in a local folder as an alternative to downloading ENA read sets.

### Preprocessing of sequencing reads

The first step in a sequencing workflow is the quality checking and trimming of the raw sequencing reads. Historically, FASTQC (Andrews 2010) has been used for quality checking and BBduk (Bushnell 2014) to remove adaptors and low-quality sequences (Martiny *et al.* 2022a, Munk *et al.* 2022). However, new and faster tools have appeared, such as the widely adopted tool fastp (Chen 2023). Therefore, we decided to compare the performance of fastp 0.23.2 with the combination of FASTQC and BBduk to ensure an efficient preprocessing of the raw reads.

### Mapping and alignment of reads against reference databases

To quantify ARGs and microorganisms, KMA 1.4.12a (Clausen *et al.* 2018) was used to map and align the trimmed reads to different databases. KMA uses *k*-mer seeding to increase mapping speed and is specifically made for mapping reads against redundant databases. We designed the alignment procedure to use two reference sequence databases: the PanRes collection of ARGs and the mOTUs3 database (Ruscheweyh *et al.* 2022) for microbiome profiling. Details on the choice and design of reference databases are described in later sections.

### Metagenome representation using Mash sketches

We used Mash 2.3 (Ondov *et al.* 2016) to enable comparison between large sets of metagenomes for subsequent selection and analysis by creating MinHash sketches as representatives for individual metagenomes. This allows an unbiased comparison of samples with a low constant memory footprint and a short turnaround time, which can be used for subsequent clustering and identification of closely related metagenomes (Ondov *et al.* 2016). We identified appropriate sketch and *k*-mer sizes using a selection of 72 sewage metagenomes: 36 from Copenhagen sewage (Brinch *et al.* 2020), 18 from various sites in the world (Munk *et al.* 2022), and the remaining 18 were technical replicates of a single sewage sample taken in Copenhagen, Denmark (PRJEB63576). Sketches were created for all samples using sketch sizes of $10^{3}$, $10^{4}$, $10^{5}$, and $10^{6}$, with *k*-mer sizes of 16, 21, 27, and 31. Mash distances were calculated to find the parameters resulting in lower within-sample distances than between-sample distances. Distances were also clustered using Dynamic Neighbor-Joining with CCPhylo 0.8.3 (Clausen 2023) to verify the appropriate sub-clustering of samples.

### Building flanking regions around genes of interest with ARGextender

To examine the genomic content surrounding ARGs, we created ARGextender to build genomic flanking regions around identified ARGs. ARGextender recruits reads using a recursive approach with KMA and SPAdes (Prjibelski *et al.* 2020), which produce comparable results and are faster than full metagenome *de novo* assembly with SPAdes. A more detailed description of ARGextender can be found in a later section. Because KMA will also assign reads to low abundant sequences that are unlikely to form contigs, we created a filtering step using the KMA mapstat files so that only samples fulfilling the following criteria would be assembled: >90% query identity, >90% global consensus identity, and a mean read depth >6.

### The PanRes database

Bacterial genes that encode resistance to antibiotic drugs, heavy metals, and biocides have been previously identified and compiled into several databases (Alcock *et al.* 2023, Bonin *et al.* 2023, Bortolaia *et al.* 2020, Feldgarden *et al.* 2021, Gupta *et al.* 2014, Gschwind *et al.* 2023) . We sought to collect these genes of interest into a single unique collection that we named PanRes; short for the pan resistance, as having a single, although highly redundant collection is computationally more efficient to search through.

The following gene databases were identified and used as seeds for the selection of genes for the PanRes collection: ResFinder (downloaded 20 January 2023, Bortolaia *et al.* 2020), ResFinderFG (version 2.0, Gschwind *et al.* 2023), CARD (version 3.2.5, Alcock *et al.* 2023), MegaRes (version 3.0.0, Bonin *et al.* 2023), AMRFinderPlus (version 3.11/2022-12-19.1, Feldgarden *et al.* 2021), and ARGANNOT (V6_July2019, Gupta *et al.* 2014). In addition to these collections, we used a set of cloned, functionally determined ARGs from environmental and clinical samples tested for resistance to antibiotics not yet on the market (Daruka *et al.* 2023). We received these sequences in November 2022 [Csaba Pál and Zoltán Farkas (Personal communication, July 2022)], and tagged them with “CsabaPal” in the collection.

From the CARD database, the genes based on the protein homolog model were included as they are acquired and do not rely on mutations for resistance. In the MegaRes gene collection, sequences with the “RequiresSNPConfirmation” tag were excluded from consideration, as these represent mutated versions of housekeeping genes, regulators, repressors, and promoter sequences. From the AMRFinderPlus sequences of the “AMR” type, only those satisfying the “AMR” subtype were used. For the genes of the “STRESS” type, just the “BIOCIDE” and “METAL” subtypes were retained.

As heavy metals often co-select for antibiotic resistance (Baker-Austin *et al.* 2006), we did a screening for metal resistance genes. The BacMet v1.1 collection of experimentally verified resistance proteins was used as a starting point (Pal *et al.* 2014), where the BacMet GenBank accessions were used to extract the coding sequences (NCBI Resource Coordinators 2018). We then manually curated the collection of metal resistance genes with sequences identified in the published literature, with a special focus on acquired cobalt, zinc, cobalt, copper, arsenite, mercury, cadmium, lead, or silver-resistance genes retrieved using the NCBI nucleotide database (NCBI Resource Coordinators 2018). The final collection of metal resistance genes we refer to as the MetalResistance database is deposited at: https://doi.org/10.5281/zenodo.8108201.

All retrieved sequences were clustered using Usearch 11.0.667 (Edgar 2010) with the fastx_uniques algorithm to identify unique sequences to include in PanRes. These unique ARGs were clustered based on 90% identity and 90% coverage with the cluster_fast algorithm. GeneAssimiliator (https://github.com/genomicepidemiology/gene\_assimilator) was used to perform this iterative approach of recruiting, clustering, and refining gene collections of various sources into one.

### ARGextender

Despite the value provided through metagenomic *de novo* assemblies, the computational demands are often too high to be considered for routine use (Martiny *et al.* 2022b). To enable a shorter turnaround time of metagenomic assemblies with lower computational demands, we developed ARGextender to perform *de novo* assemblies around target sequences of interest.

ARGextender recursively applies KMA 1.4.12a (Clausen *et al.* 2018) and SPAdes 3.15.5 (Nurk *et al.* 2017, Prjibelski *et al.* 2020), where KMA is used to identify the target sequences in the sample, followed by a *de novo* assembly of the reads matching the target(s) using SPAdes. After each *de novo* assembly, scaffolds containing target sequences are extracted using KMA and set as the new target. This recursion is repeated until no more reads are included in the *de novo* assembly or the user-defined maximum number of recursions has been met (unlimited by default). When the targeted *de novo* assembly has saturated, the scaffolds and assembly graph are saved, along with a table containing the information about which target sequences are found within each scaffold. These include target sequences with an alignment score within 70% of the best-scoring target sequences.

We evaluated the ARGextender tool by comparing the output of ARGextender to full *de novo* metagenomic assemblies of 951 urban sewage samples published by (Munk *et al.* 2022). We compared the resulting scaffolds by matching the sequences of the ResFinder database to the scaffolds using KMA 1.4.12a with the “-ont” parameter. The surrounding flanks were then extracted and compared.

### Evaluating tools for profiling microbiomes

We compared the performance of KMA (Clausen *et al.* 2018) with several microbial reference databases and profiling tools. Using the *in silico* data generated for the profiling test in the Critical Assessment of Metagenome Interpretation (CAMI) challenge (Meyer *et al.* 2022), we tested mOTUs 3.0.3 (Ruscheweyh *et al.* 2022), MetaPhlAn 4.0.3 (Blanco-Míguez *et al.* 2023), Bracken 2.8 (Lu *et al.* 2017) on Kraken2 (Wood *et al.* 2019) output, and KMA 1.4.7 with the following reference databases: the mOTUs database (Ruscheweyh *et al.* 2022) of 521 780 sequences (version 3.0.1, downloaded 5 December 2022), the Silva database (Quast *et al.* 2012) of 2 225 272 sequences (version 138, downloaded 16 January 2020), and a custom database of the genomic database of 16 732 113 genomic sequences (downloaded 24 May 2022, compiled using a previously described procedure (Osakunor *et al.* 2020).

The performance of each tool and reference database was evaluated similarly to the CAMI challenge using the OPAL tool (Meyer *et al.* 2019). Results with low abundances were filtered away (“-f 1”). We used three binary classification metrics on each taxonomic level, from superkingdom to species, to determine performance and the sum of abundances, specifically purity, completeness, and F1 scores. Purity and completeness consider the performance of correctly identifying taxons without considering relative abundances, where TP is the number of true positives, FP is the number of false positives, and FN is the false negatives: Purity=TP/(TP+FP) and Completeness=TP/(TP+FN). The F1 score measures the overall performance of taxon identification and is defined as:

$$\begin{array}{c} F1\;\text{score}=2\frac{\text{purity}\cdot \text{completeness}}{\text{purity}+\text{completeness}} \end{array}$$

## Code and data availability

The ARGprofiler pipeline is available on GitHub, at https://github.com/genomicepidemiology/ARGprofiler under the APACHE-2.0 license. ARGextender is included as part of the ARGprofiler repository. GeneAssimilator is available at https://github.com/genomicepidemiology/gene_assimilator. The code for the comparison of microbial profilers is available at https://github.com/genomicepidemiology/argprofiler-tax-benchmark. Jupyter Notebooks supporting the various figures and tables have been shared at https://github.com/genomicepidemiology/argprofiler-paper-notebooks.

The MetalResistance gene database is available on Zenodo, at https://doi.org/10.5281/zenodo.8108201, and the first version of the PanRes collection is available at https://doi.org/10.5281/zenodo.8055115. Output files of benchmarking microbial profilers are available at https://doi.org/10.5281/zenodo.7923774. The full de novo assemblies of urban sewage samples are available on ENA under project accessions PRJEB40798, PRJEB40816, PRJEB40815, PRJEB27621, and ERP015409. The Copenhagen sewage collection is under PRJEB34633, and the repeated resequencing of a single Copenhagen sewage sample is under PRJEB63576.

### Results

With ARGprofiler, we wanted to focus on creating a pipeline that produces three main outputs suitable for analyzing metagenomic datasets: the abundance of reads aligned to different genes of two suitable reference databases (mOTUs and PanRes), targeted *de novo* assemblies with ARGextender, and MinHash sketches with Mash (Fig. 1). Our pipeline has been designed to be suitable for large volumes of sequencing reads by using the workflow manage Snakemake and by carefully selecting appropriate tools.

### The PanRes collection

**Figure 2.**
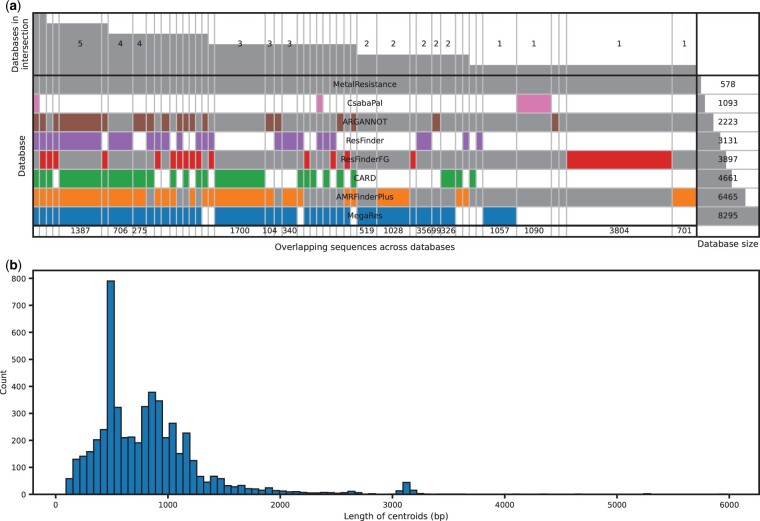
Overview of the sequences included in PanRes. (a) A comparison of overlaps between the different databases in the PanRes collection. (b) Distribution of antimicrobial resistance gene lengths.

PanRes was created to compile several existing ARG reference databases into one, as there are both overlaps and discrepancies between the different ones (Fig. 2a). Out of 30 400 genes, we identified a set of 14 078 unique sequences that were included in PanRes. Grouping these genes based on 90% identity and 90% coverage produced 5280 centroids, which ranged in lengths between 93 and 5972 bp, with a median of 762 bp (Fig. 2b).

### Assembling flanks around genes with ARGextender

**Figure 3.**
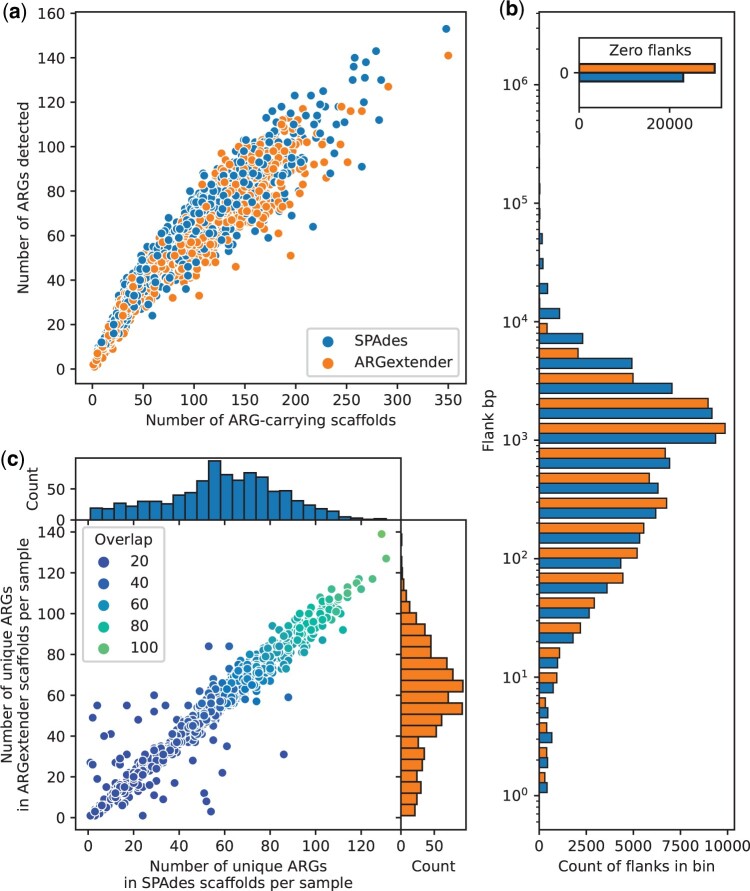
A comparison between the flanks extracted around ARGs found in scaffolds produced with either SPAdes or ARGextender. (a) Number of scaffolds containing at least one ARG compared with the number of ARGs detected across all scaffolds in a sample. (b) Distribution of flanking content (bp: basepairs) in sewage samples, including the number of scaffolds without a flanking region in the top right corner. (c) Overlap between the tool regarding which ARGs had flanks, excluding the scaffolds with zero flank regions. Only ARGs with a minimum of 95% breadth of coverage were included in this figure. ARG: antimicrobial resistance genes.

To validate the output of ARGextender, we compared the scaffolds with those produced by SPAdes on a set of 951 urban sewage samples (Fig. 3). On average, ARGextender built 101 scaffolds (range: 1–350), whereas SPAdes reported 95 ARG-scaffolds on average (range: 1–348). The number of distinct ARGs detected in the scaffolds was on average 57 for ARGextender (range 1–141) and 58 for SPAdes (range: 1–153) (Fig. 3a). Most of the flanks extracted around ARGs were between 100 and 5000 bp, although many had no flanking regions (Fig. 3b). Excluding scaffolds with zero flanks, we can see that ARGextender and SPAdes can assemble flanks around a similar number of ARGs (Fig. 3c). Overall, ARGextender was capable of assembling the same amount of flanks as SPAdes with lower computational requirements (Supplementary Material Appendix B), although SPAdes produced longer flanks for some samples.

### Choosing a microbial profiler and reference database

**Figure 4.**
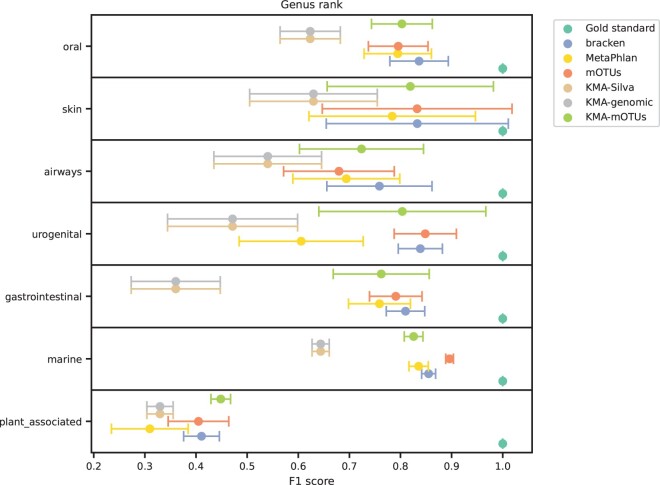
Performance of microbial profilers on the *in silico* CAMI data as measured by the F1 score at genus rank. The harmonic mean is reported as the circle, and the error bars are the standard deviation. CAMI: critical assessment of metagenome interpretation.

Since there are multiple different tools and reference databases available to profile the microbial content of a sample, we compared the performances of each method with various databases (Supplementary Material Appendix A, Fig. 4). KMA reported more matched reference sequences, regardless of the database used, where most were false positives (Supplementary Material Fig. A1). After removing hits with low abundances, KMA were comparable with MetaPhlAn and mOTUs, sometimes identifying more taxons than the other tools (Supplementary Material Fig. A2). We observed a decrease in completeness and purity in taxon identification for the non-human environments for all tools and databases (Supplementary Material Figs A3 and A4). Despite this decrease, KMA had higher completeness than MetaPhlAn, mOTUs, and Bracken on the plant-associated samples (Supplementary Material Fig. A4a). KMA with the mOTUs sequences (KMA-mOTUs) outperformed KMA with genomic and Silva databases across all six sampling groups regarding binary metrics (Supplementary Material Appendix A). While KMA-mOTUs abundance results were generally lower, the F1 scores were on par with the MetaPhlAn and mOTUs profilers (Supplementary Material Figs A5 and A6), which we believe is due to a difference in how abundance is calculated in the other tools.

We decided on combining KMA with the mOTUs sequences for two reasons. First, we plan to apply our pipeline as an environment-agnostic passive surveillance tool encompassing many One Health settings important for ARG ecology. Therefore, we deemed performance outside just human microbiomes important, where KMA-mOTUs outperformed all other tools on the plant-associated samples and were on par with marine samples (Fig. 4). Second, since we also use KMA for the ARG quantification, this choice limited the overall pipeline complexity.

### Clustering and representing metagenomes using Mash sketches

Most of the output of ARGprofiler relies on known reference sequences. To enable unbiased metagenome-wise comparison and clustering of sequence runs, we include the creation of sketches using Mash. We tested the discriminatory power of different sketch and *k*-mer sizes on sewage samples, some of which were technical sequence replicates of the same sample. Both the very small sketch sizes and short *k*-mers failed to clearly distinguish between technical replicates of the same sample and sequence runs of other sewage samples (Supplementary Material Fig. B5 in appendix). Technical replicates were efficiently separated from the remaining samples with a *k*-mer size of 31 and sketch size of $\geq 10^{4}$ (Supplementary Material Fig. B6 in appendix). As smaller sketch sizes require less computational resources, a sketch size of 104 was included as default in ARGprofiler.

### Reducing computational time and memory usage

Analyzing the large quantities of metagenomic data currently available at ENA and future data is not computationally trivial, and choosing efficient workflows will seriously impact the associated time, costs, and energy expenditure. We did a benchmark of each rule using a set of metagenomic datasets from a variety of sampling origins (details in Supplementary Material Appendix B), where we observed that with our final parameter settings, the ARGprofiler pipeline processed 1.21 gigabasepairs/h (Gbps/h) with a median processing performance of 0.36 Gbps/h. Most steps had a sample-average peak memory footprint below 1 GB and required less than a CPU hour, except for KMA-mOTUs and ARGextender (Table 1).

**Table 1.** Measured times and memory usage for each step of the ARGprofiler pipeline.

Rule	Tool	CPU time (h)		Memory (MB)	
		μ	σ	μ	σ
Read download	fastq-dl	0.58	1.33	1389.83	1052
Read preprocessing	fastp	0.51	1.23	2375.95	1748.43
Microbiome read alignment	KMA-mOTUs	2.66	6.04	6817.09	3.44
ARG read alignment	KMA-PanRes	0.43	0.99	308.75	137.05
Building flanking regions	ARGextender	2.06	5.20	539.61	804.92
Creating MinHash sketches	Mash sketch	0.24	0.59	2.03	0.10

The average (μ) CPU time in hours (h) and the peak memory in megabytes (MB) are reported together with standard deviations (σ). Note that the CPU hours and peak memory for ARGextender did not include the two samples that were not completed within our limit of 48 h.

ARG: antimicrobial resistance genes.

## Discussion

There are currently terabytes of metagenomic sequencing data available in public databases, and producing standardized and consistent results is necessary for downstream analyses. Therefore, we have designed ARGprofiler to allow efficient ARG-monitoring and quantification in vast amounts of sequencing data, determine flanking regions around ARGs for downstream epidemiological investigation, and *k*-mer-based comparison of sequence runs. Each output aligns with our overall goal of reanalyzing public sequencing datasets for the characteristics of ARGs in a global microbial and environmental context.

One of the unique features of ARGprofiler is the addition of the PanRes database. The motivation behind PanRes was to eliminate the inefficiency associated with searching for the same gene in multiple collections and the additional overhead of spawning extra compute jobs for each collection. We, therefore, sought to collect the unique sequences of ARGs from a wide spectrum of existing databases. It is our hope that this will help facilitate fewer but larger and more efficient monitoring runs of public metagenomes, followed by data sharing and the individual AMR researchers then filtering results to their specific focus.

An important feature of ARGprofiler is the creation of targeted assemblies around genes with ARGextender. ARGextender uses KMA and SPAdes to create targeted *de novo* assemblies by identifying if the targeted ARGs are present in a sample and then recruiting reads to the surrounding regions. The pairwise comparison of ARG-carrying scaffolds produced with ARGextender and SPAdes in sewage samples showed that ARGextender could extract comparable flanking regions to SPAdes but in a much shorter time frame. This approach also avoids running the more expensive algorithm if none of the target genes is identified.

However, there are still a few points we need to address in the way that ARGprofiler currently works. First, ARGprofiler is designed to only work with short-read sequencing data, thus not utilizing the advantages of long-read sequencing technologies. We are planning to extend the input options to include long-read datasets. Second, a significant aspect of ARGprofiler is the choice of reference databases. PanRes is a one-size-fits-all approach for ARGs. However, as it combines different sources and scopes, it will be up to the individual research questions, which subsets are appropriate for consideration. ARGprofiler also profiles the microbiome of each sequencing dataset by mapping the reads against the microbial reference sequence database mOTUs. This step is included to compare the abundance of ARGs to the microbial content (Martiny *et al.* 2022b, Munk *et al.* 2022, Johansson *et al.* 2023). We chose mOTUs as this collection performed best across different environments with KMA. Our choice was based on the *in silico* datasets, and while real data might contain many more unknowns, it appeared to be the best choice for our pipeline. We chose to incorporate a sub-workflow of creating Mash sketches with optimized parameters to allow the user to compare and cluster to determine similar sequencing datasets and those of poor quality. The sketches also make it possible to query the read sets against pre-sketched genomes, thus allowing the user to re-use the data without rerunning the whole pipeline.

In conclusion, we have implemented and evaluated ARGprofiler to be a robust bioinformatic pipeline that provides other researchers the opportunity to analyze large collections of metagenomic sequencing runs against a collection of ARGs or other genes of interest. The ARGprofiler code is publicly available under the Apache-2.0 license at https://github.com/genomicepidemiology/ARGprofiler.

## Supplementary Material

btae086_Supplementary_Data
