# Supplementary material for: ARGprofiler—a pipeline for large-scale analysis of antimicrobial resistance genes and their flanking regions in metagenomic datasets
Source: Bioinformatics. 2024 Feb 20;40(3):btae086. doi: 10.1093/bioinformatics/btae086 (PMC10918635; doi:10.1093/bioinformatics/btae086)
Supplement: btae086_Supplementary_Data [file btae086_supplementary_data.zip › Appendix A_Benchmarking_different_taxonomic_profilers.pdf]

Benchmarking different taxonomic profilers

These figures were generated based on the output of using the OPAL[1] tool with the filter set to 1 ('—filter 1'). Since we are mainly interested in the capability of the different tools to capture groups at taxonomic ranks, we have only reported the sum of abundances per rank beside the binary metrics.

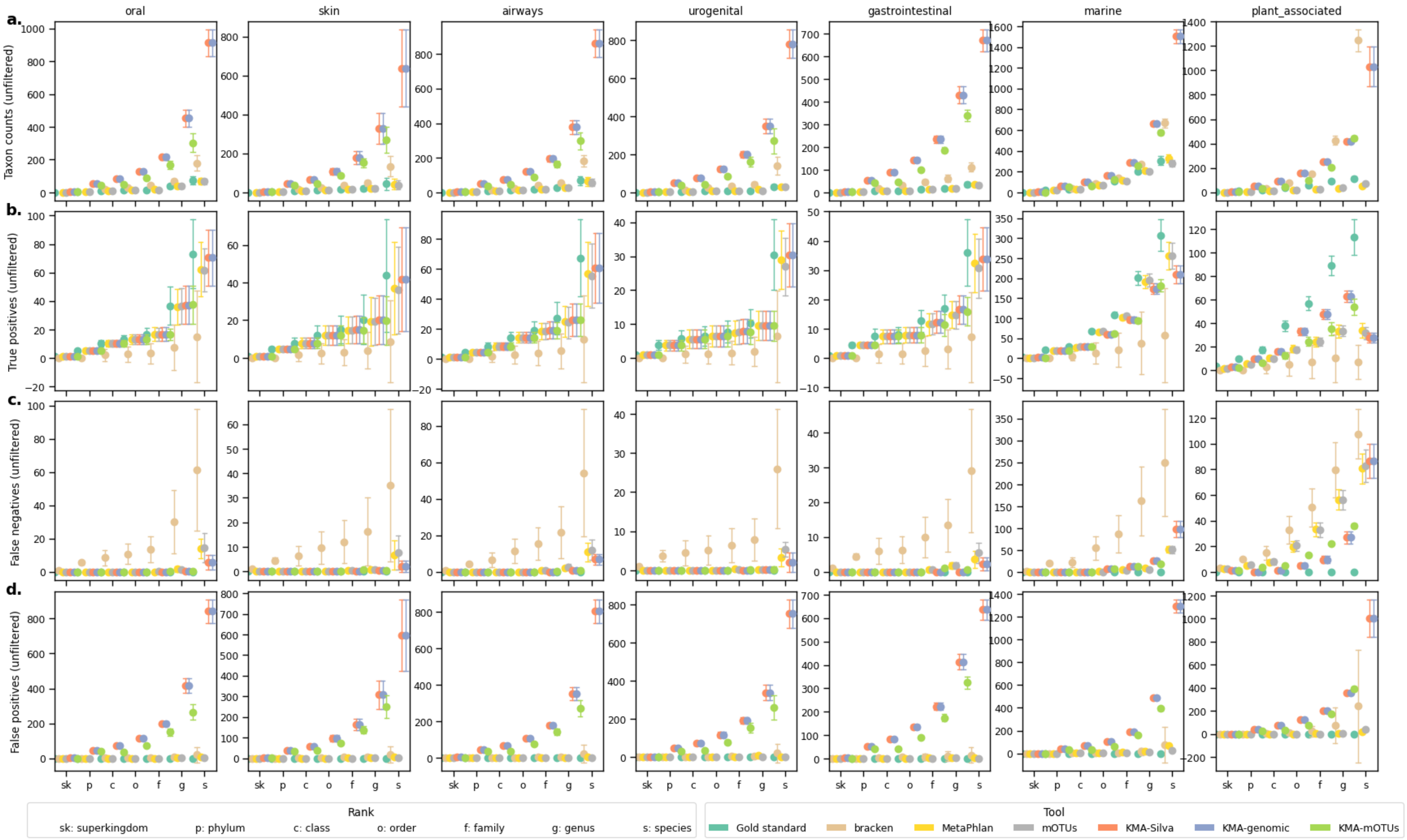

Figure A1: Overview of the number of unfiltered a. positive hits, b. true positives, c. false positives, and d. false negatives per taxonomic rank for each of the tools tested. The harmonic mean is reported as the circle and the error bars are based on the standard deviation.

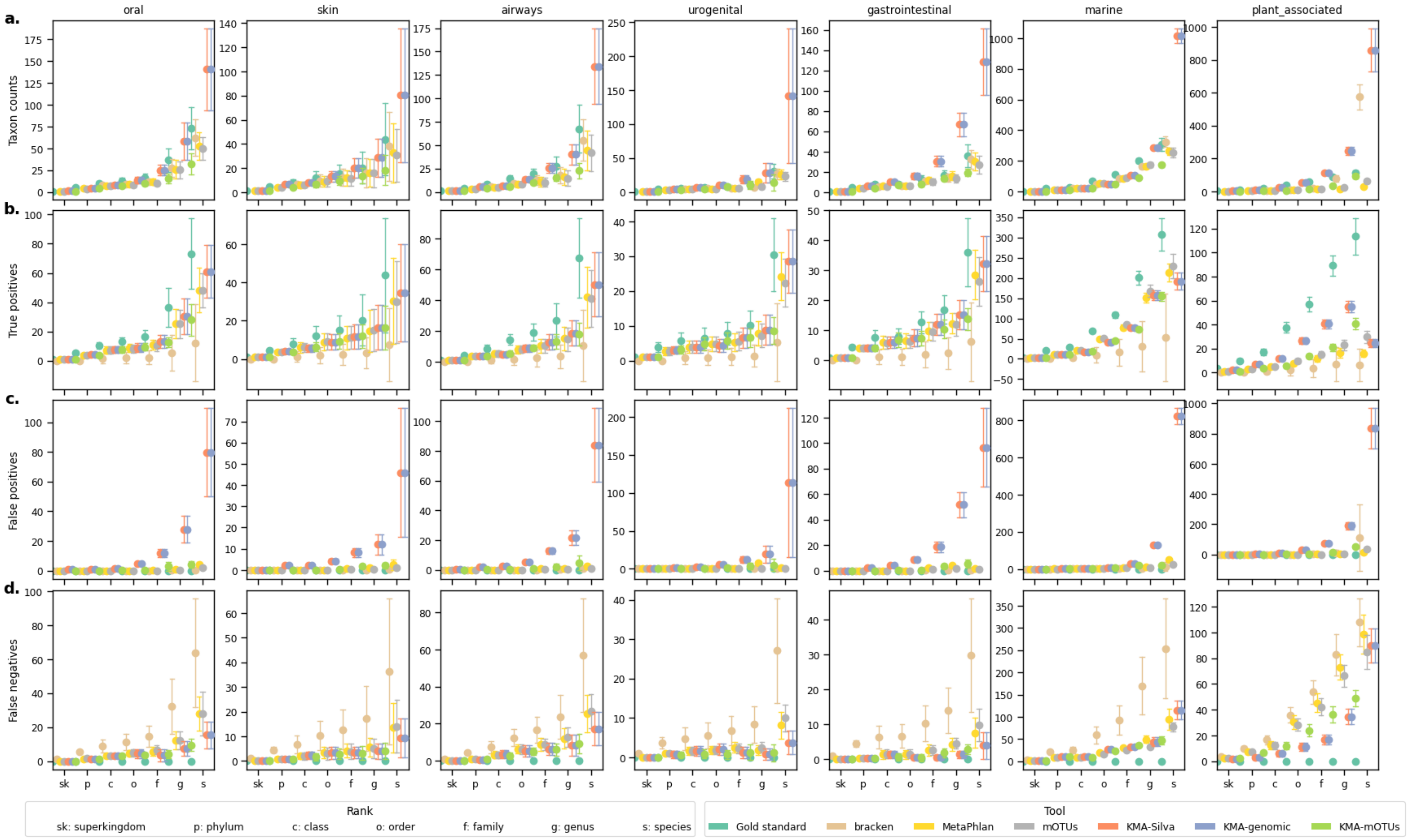

Figure A2: Overview of the number of filtered a. positive hits, b. true positives, c. false positives, and d. false negatives per taxonomic rank for each of the tools tested. The harmonic mean is reported as the circle and the error bars are based on the standard deviation.

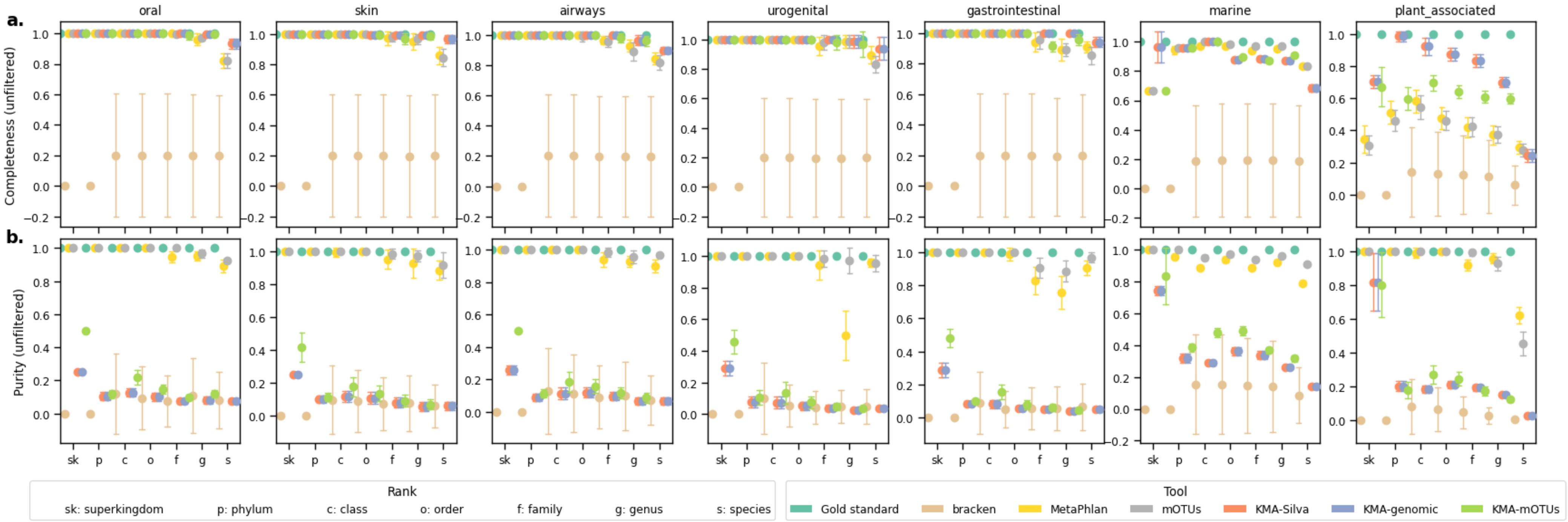

Figure A3: a. Completeness and b. purity based on unfiltered results for each tool tested across different ranks. The harmonic mean is reported as the circle and the error bars are based on the standard deviation.

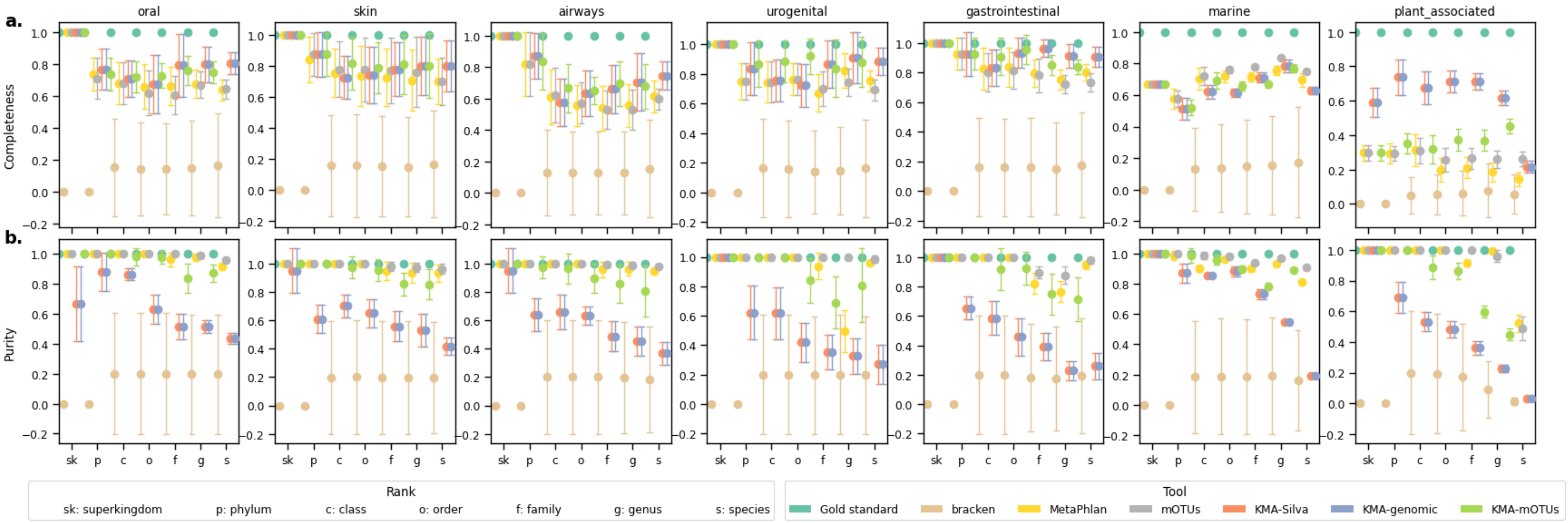

Figure A4: a. Completeness and b. purity based on filtered results for each tool tested across different ranks. The harmonic mean is reported as the circle and the error bars are based on the standard deviation.

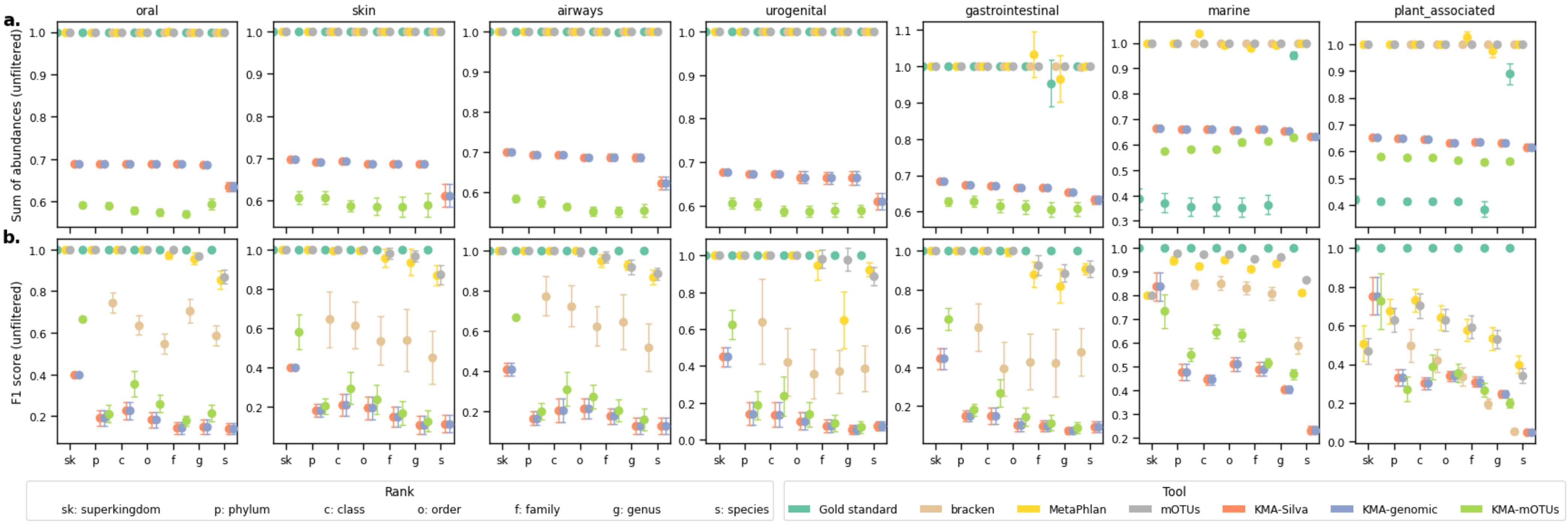

Figure A5: a. Sum of abundances and b. F1 score of unfiltered results. The harmonic mean is reported as the circle and the error bars are based on the standard deviation.

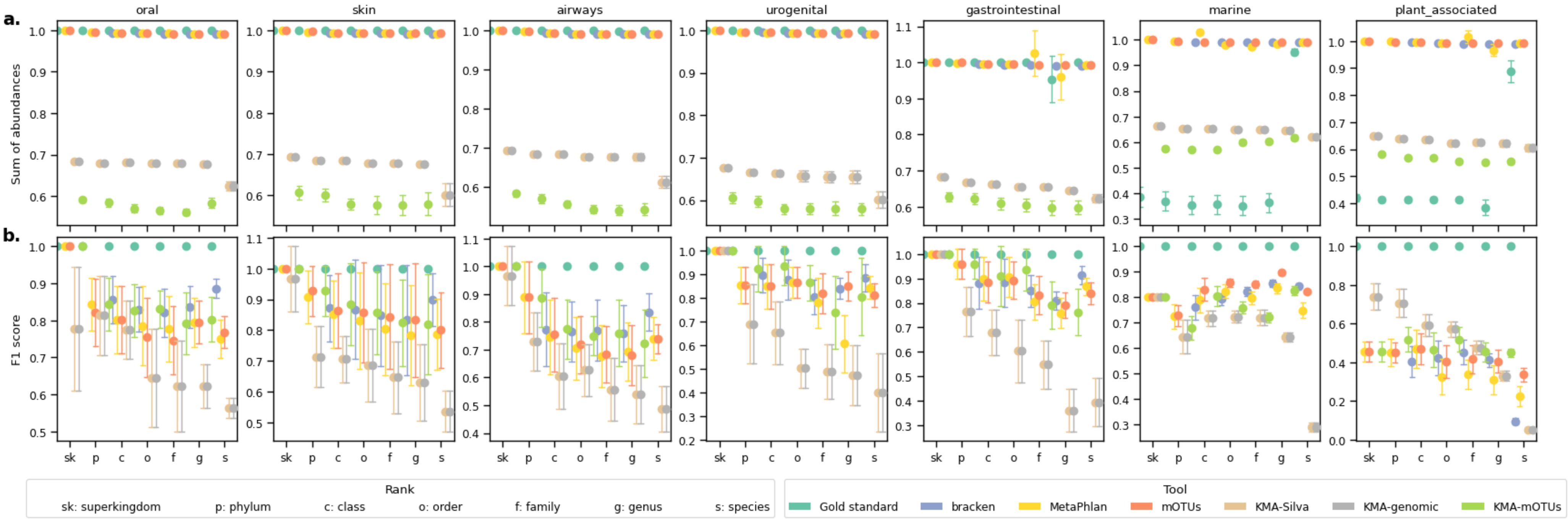

Figure A6: a. Sum of abundances and b. F1 score of filtered results. The harmonic mean is reported as the circle and the error bars are based on the standard deviation.
