# Supplementary material for: ARGprofiler—a pipeline for large-scale analysis of antimicrobial resistance genes and their flanking regions in metagenomic datasets
Source: Bioinformatics. 2024 Feb 20;40(3):btae086. doi: 10.1093/bioinformatics/btae086 (PMC10918635; doi:10.1093/bioinformatics/btae086)
Supplement: btae086_Supplementary_Data [file btae086_supplementary_data.zip › Appendix_B_assessments.pdf]

Assessing each step of the ARGprofiler pipeline

We decided to assess every rule of the ARGprofiler pipeline to optimize the tool parameters and computational requirements to produce the needed output as quickly and cost-effective as possible. We have previously analyzed 214,095 metagenomic datasets (Martiny *et al.* 2022) by aligning trimmed read fragments against ARGs of the ResFinder database (Zankari *et al.* 2012; Bortolaia *et al.* 2020) and 16S/18S rRNA genes of the Silva database (Quast *et al.* 2013). Based on the number of trimmed fragments and the number of those fragments aligned to ARGs (Figure B1), we selected 29 different metagenomic sequencing datasets that represented a diverse set of sampling origins and outcomes of our alignment procedure (Table B1).

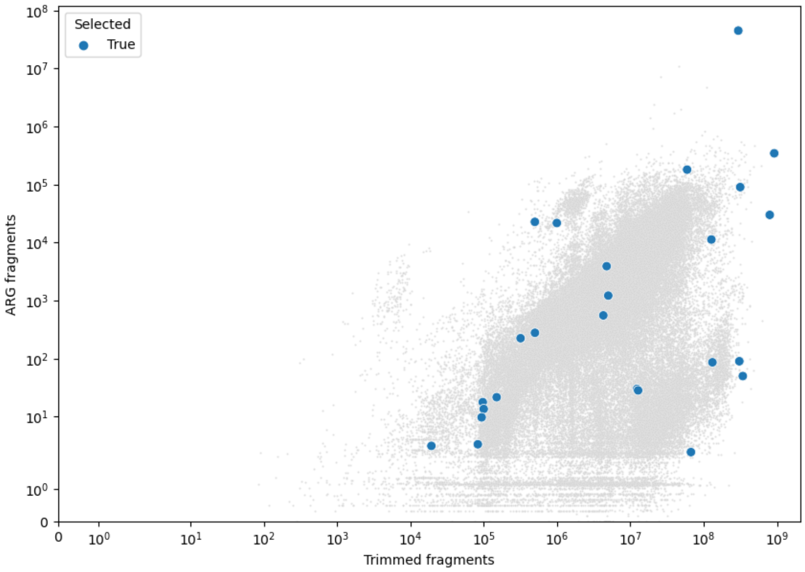

Figure B1. The 29 datasets we selected for benchmarking in relation to the other 214K metagenomic datasets in terms of the number of trimmed fragments (x-axis) and the number of fragments aligned to ARGs (y-axis). The figure is reproduced from Martiny (Martiny et al. 2022) *and data is available at https://doi.org/10.5281/zenodo.7553776.*

					Shannon		Simpson		Aligned fragments	
					ARG	Genera	ARG	Genera	ARG	Genera
**run_accession**	**host**	**raw_reads**	**raw_bases**	**trimmed_ fragments**						
ERR056997	Homo sapiens	154131	126490081	149846	1,25537	0,556237	0,60036	0,206514	21,6072	316,093
ERR1135612	pig gut metagenome	500074	40153320	317694	2,88256	3,57557	0,90902	0,950361	224,706	204,3
ERR1884262	unidentified	148385427	12609703551	131474674	1,16796	4,93197	0,506904	0,96308	86,3054	12620,2
ERR209998	Homo sapiens	168799	9778565	19285	1,24365	2,21383	0,980444	0,985053	3,13192	9,25777
ERR2239761	Homo sapiens	500015	75409030	498795	2,66422	2,8953	0,891401	0,901413	278,396	449,107
ERR262503	Homo sapiens	3450	434671	3432	-	3,33488	-	0,944661	-	1548,82
ERR2764819	Homo sapiens	5000861	860431392	4990010	2,40144	3,11016	0,808635	0,918955	1219,77	7910,15
ERR299538	Phytophthora infestans	558444701	52493801894	341583763	2,29623	3,5438	0,900126	0,936741	50,008	7329080
ERR3213717	metagenome	100034	342607177	96762	2,12539	2,50524	0,907243	0,976611	17,7836	13,5261
ERR3593315	gut metagenome	59595430	17878629000	59386874	4,3301	2,9719	0,978211	0,911869	180885	37467,1
ERR3641954	gut metagenome	793116618	119760609318	314917681	2,9441	3,26143	0,902678	0,939748	90390,3	227171
SRR10154308	Homo sapiens	125764	11318192	60497	-	-	-	-	-	0,665779
SRR10158848	Homo sapiens	927822438	278346731400	916901366	2,50357	3,18836	0,845508	0,917001	346056	293302
SRR1027651	activated sludge metagenome	1351844737	273072636874	796229684	3,70158	5,16774	0,944582	0,982801	30010,2	97755,8
SRR1044677	freshwater metagenome	500920	251461840	497536	0,728926	1,02746	0,488392	0,398249	22748	1256,41

SRR1179181	aquatic metagenome	584783166	59063099766	306171727	2,27772	4,60492	0,824027	0,946743	90,1036	45492,6
SRR1524512	indoor metagenome	100156	50278312	99738	1,93298	3,13682	0,90336	0,956803	13,5416	51,3213
SRR2094831	marine metagenome	2189641	490479584	149	-	0	-	0	-	1,41443
SRR2237446	oral metagenome	100069	20213938	82915	1,31641	3,02427	0	0,869817	3,33558	76,3904
SRR3434587	Meleagris gallopavo	129153983	26089104566	127258030	2,56566	2,05208	0,899386	0,692599	11304,7	51212,4
SRR3989182	marine metagenome	1000432	300129600	992092	0	0,179442	0	0,0522962	21743,9	1008,77
SRR4929941	food metagenome	5130293	771380970	4271442	0,0145882	0,761608	0,00405097	0,214556	555,852	7409,39
SRR5950773	Homo sapiens	10001707	1000023109	4744619	1,20163	3,51083	0,509893	0,98711	3915,68	39,5111
SRR6401741	marine metagenome	67110021	16777505250	66987307	0,692943	4,83687	0,845083	0,971733	2,44748	4832980
SRR7125621	Homo sapiens	100101	30099486	93447	2,09216	3,4209	0,963543	0,948665	9,72456	509,063
SRR7533096	Panda	318674894	31867489400	296279879	0,182657	1,19488	0,0443512	0,643077	45058500	6149840
SRR7992574	Homo sapiens	13782136	3473098272	12347266	1,80073	4,19086	0,826479	0,942973	30,1271	3245450
SRR7992575	Homo sapiens	14276353	3597640956	12746108	1,87237	4,19718	0,870425	0,943704	28,3129	3364210
SRR9178241	marine metagenome	15811355	4747984009	3204034	-	3,16615	-	0,881587	-	274,092

Table B1. Overview of 29 samples the different tools was benchmarked on. The aligned fragment counts are adjusted by the length of reference sequences in kilobases and then summed together for the category. Data can be retrieved from https://zenodo.org/record/7553776.

Download of raw sequencing reads from ENA

Using enaDataGet from the enaBrowserTools toolkit 1.1.0 (https://github.com/enasequence/enaBrowserTools), it takes a maximum of a quarter of an hour to download the chosen datasets requires the same amount of memory each time (Figure B2).

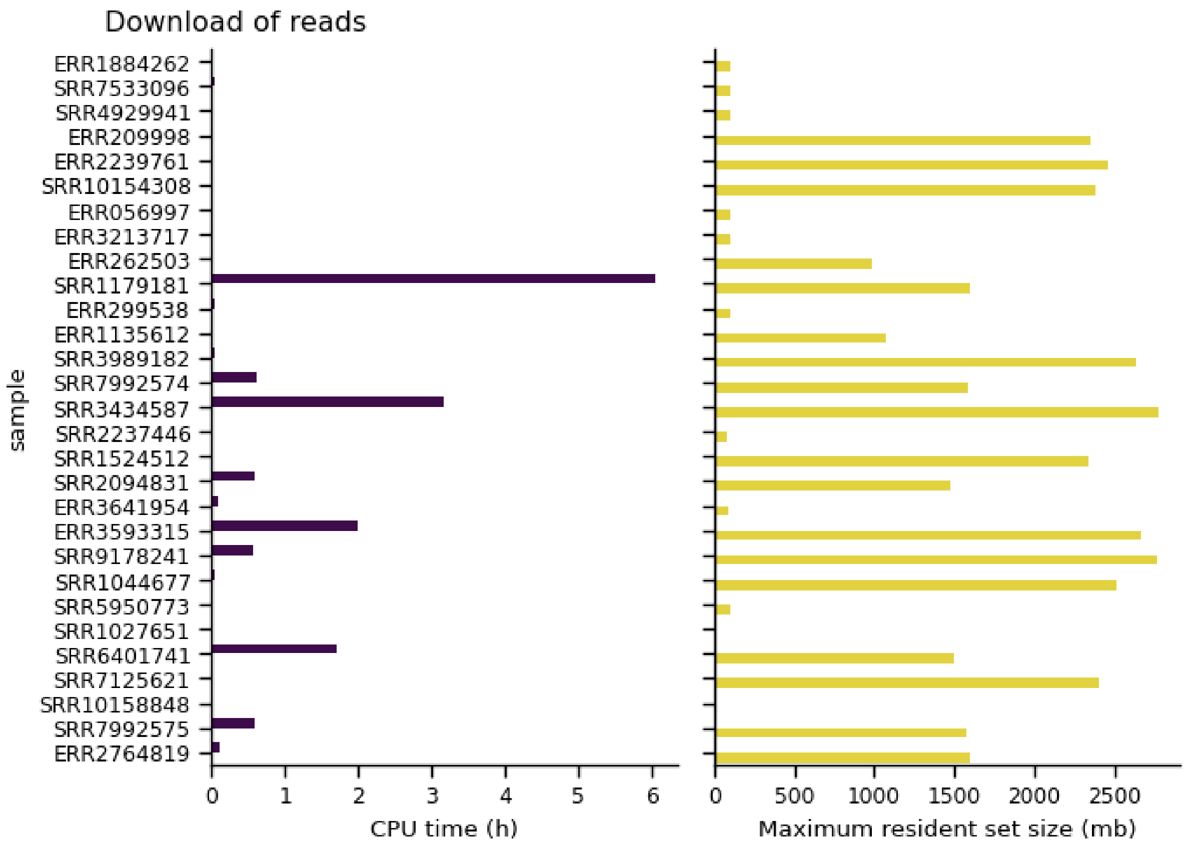

Figure B2. Time (hours) and memory (megabytes; mb) requirements for download of sequencing datasets from ENA.

Trimming sequencing reads

The first major step of the ARGprofiler pipeline is to produce trimmed reads, where we have previously used BBduk (Martiny *et al.* 2022). However, upon inspecting the time required for BBduk (Bushnell 2014), we discovered that there seemed to be a major bottleneck in the zipping and unzipping of the reads (Figure B3a). When we changed bbduk not to produce zipped trimmed reads, the time requirement was reduced, but the memory increased (Figure B3b). Therefore, we decided to compare the requirements of BBduk to fastp (Chen 2023), where we saw that fastp is both faster and needs less memory than bbduk both with and without the zipping step (Figure B3c).

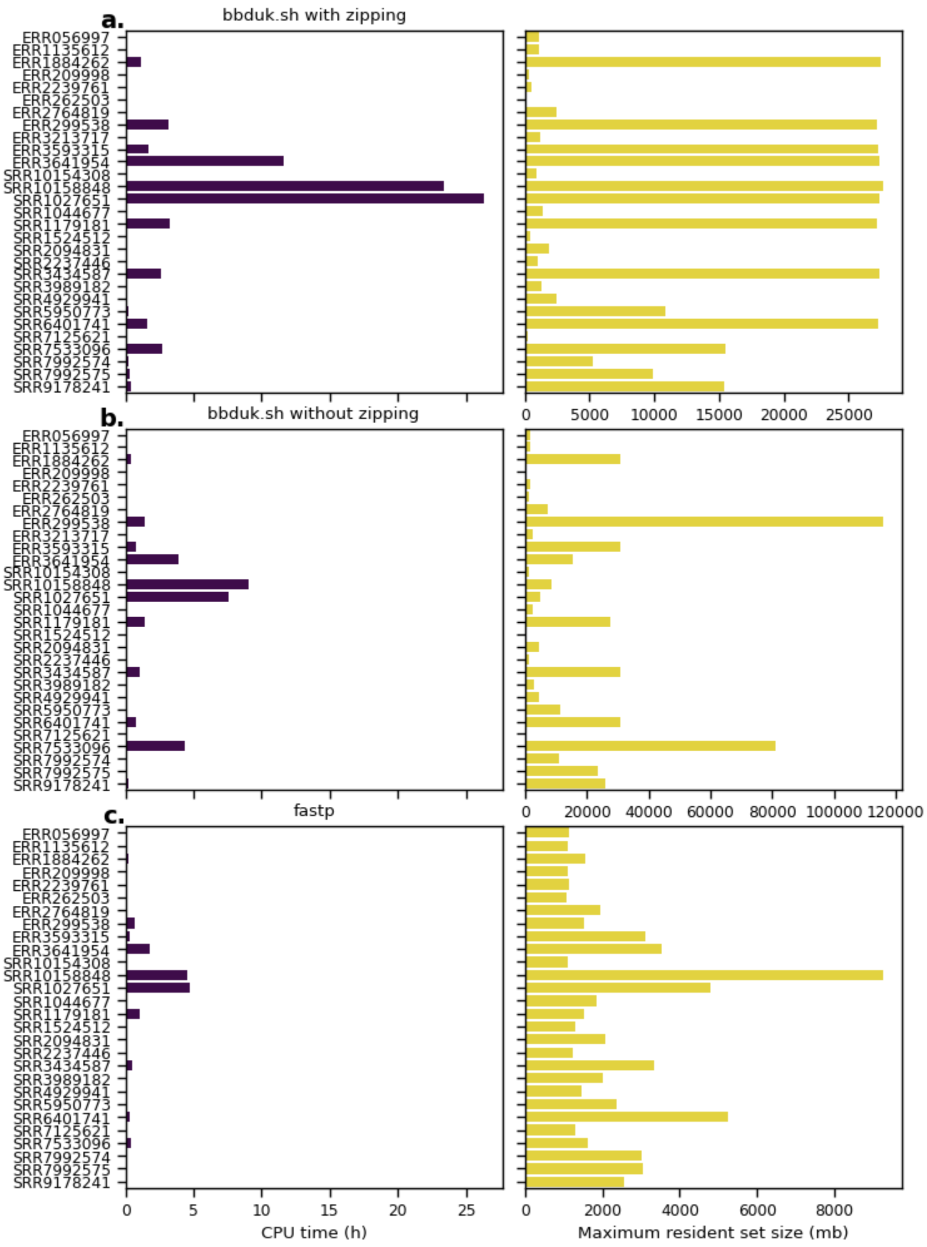

Figure B3. Time (hours) and memory (megabytes; mb) requirements for trimming reads using either a. BBduk with zipping of trimmed reads, b. BBduk without zipping of trimmed reads, and c. fastp.

Mapping and alignment of trimmed reads with KMA

Not surprisingly, it is quicker to use KMA on smaller reference sequence databases than larger ones, as illustrated with the time measurements for KMA using PanRes and mOTUs (Figure B4). However, none of the 29 runs that took more than 24 hours with KMA-mOTUs and memory usage were consistent.

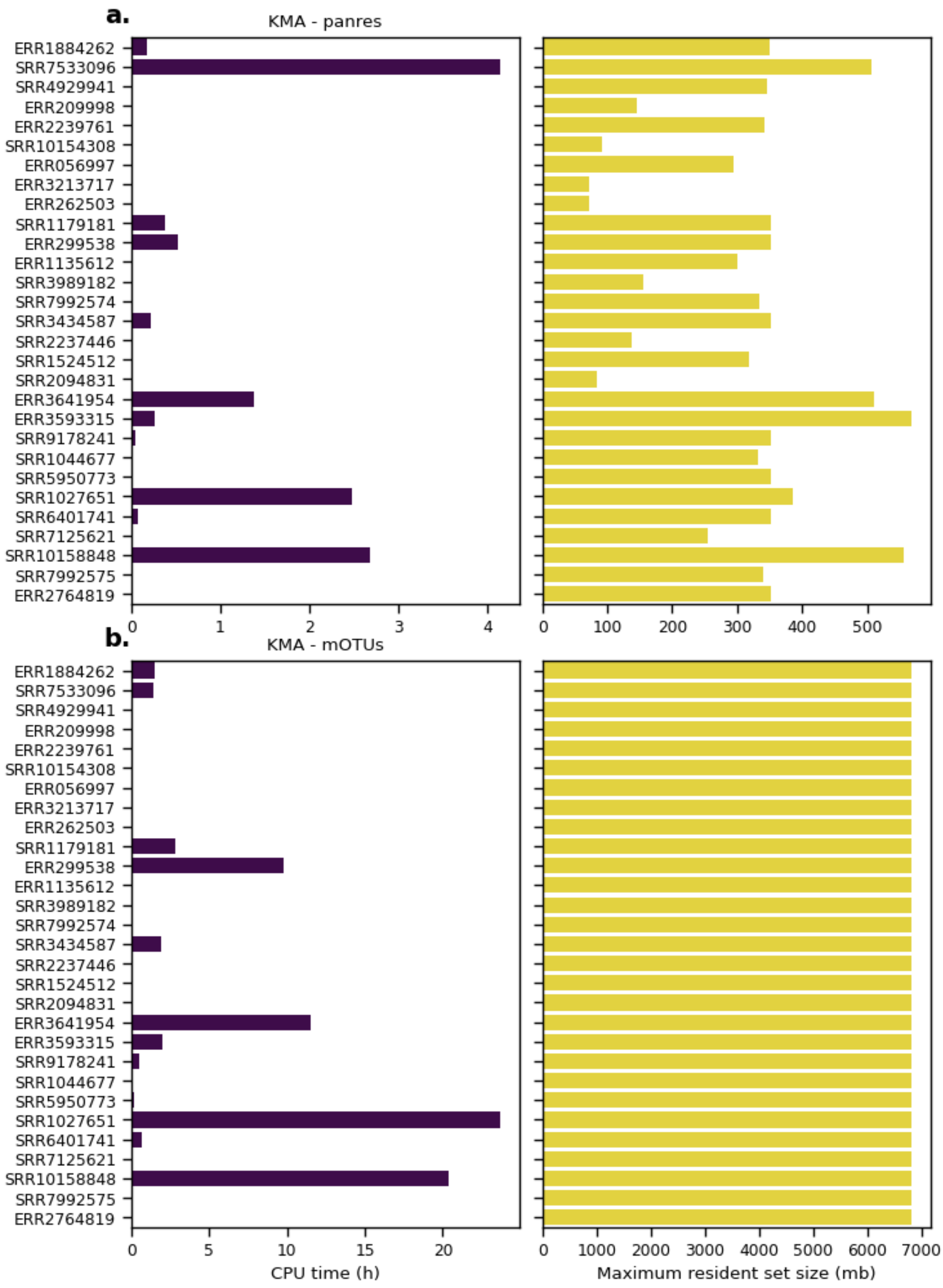

Figure B4. Time (hours) and memory (megabytes; mb) requirements for running KMA on trimmed reads using a. PanRes and b. mOTUs reference databases.

Mash sketches

To allow the comparison of genetic distances of metagenomic samples, we used Mash (Ondov *et al.* 2016) to create MinHash sketches. We wanted to identify the appropriate sketch and *k*-mer sizes so that it would be possible to distinguish between closely related samples. Therefore, we used 72 sewage samples and tested sketch sizes of 10^3, 10^4, 10^5, and 10^6 and *k*-mer sizes of 16, 21, 27, and 31. Mash distances were inferred for each

combination of sizes and clustered using Dynamic Neighbor-Joining with CCPhylo 0.8.3 (Clausen 2023).

A good set of parameters should be able to distinguish between technical sequencing replicates of the same sample and those of different sampling origins. In Figure B5, it can be seen that using a small sketch size and short *k*-mers, it was impossible to clearly distinguish between the technical replicates of one sample and the sequencing of different samples. The replicates were more distinguished with a *k*-mer size of 31 and a minimum sketch of ≥10^4. Since a sketch size of 10^4 was comparable with larger sizes (Figure B6) and required fewer computational resources, we decided to include that as a default in ARGprofiler.

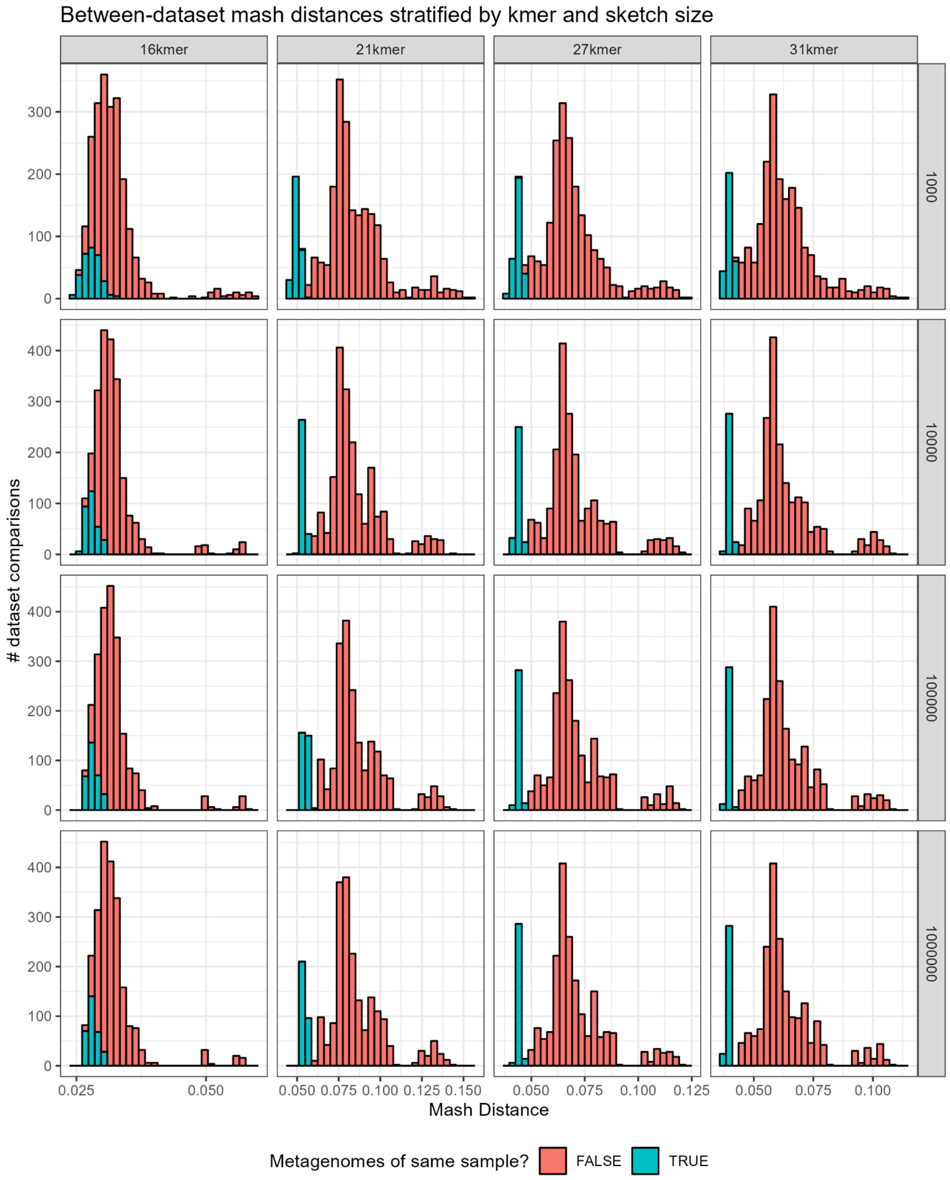

Figure B5. Distribution of Mash distances for selection of appropriate parameters for sketches. Here, the coloring indicates whether the pairwise distance is between two technical replicates of the same sample (blue) or whether the distance is between different samples (red). Columns are the k-mer sizes, and the rows are sketch sizes.

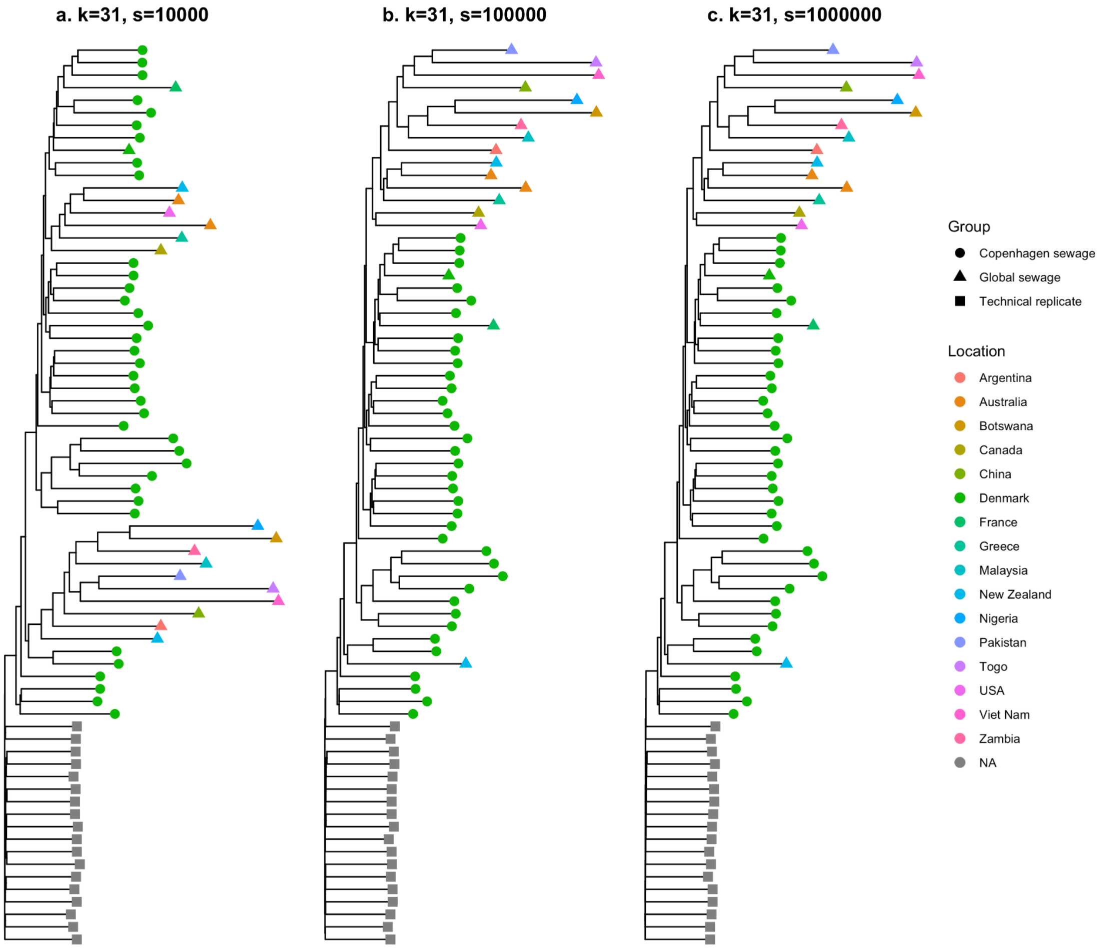

*Figure B6. Clustering of samples based on Mash distances for k-mer size 31 and sketch sizes of **a.** 10000, **b.** 100000, and **c.** 100000.*

Creating the MinHash sketches was relatively fast based on the 29 samples, as none of the sets exceeded 2.5 hours (Figure B7). That is quite beneficial as it enables the fast lookup of genes that might not be in the two reference databases we use with KMA.

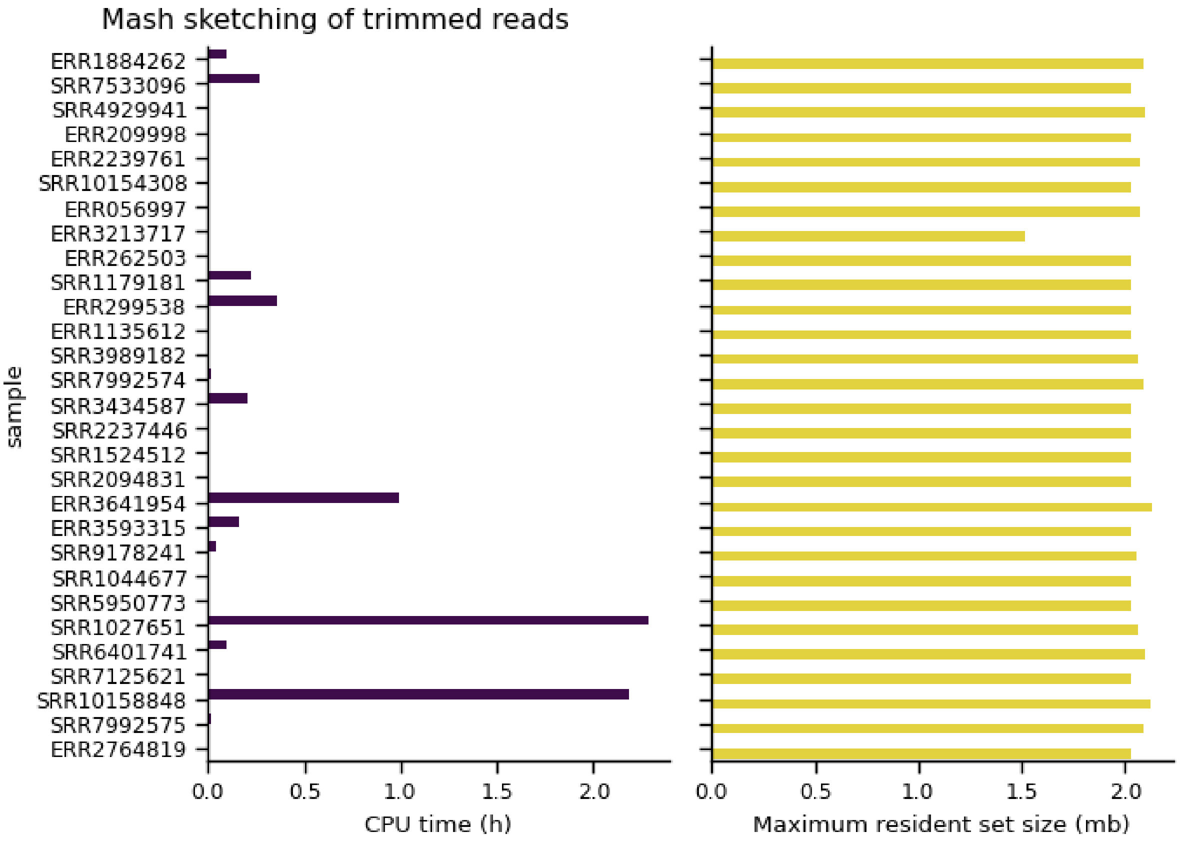

Figure B7. Time (hours) and memory (megabytes; mb) requirements for creating Mash sketches from trimmed reads.

ARGextender

We created ARGextender to assemble the flanking regions around target genes faster, which in our case is the ARGs. To validate the performance of ARGextender, we compared the output of scaffolds produced with SPAdes to those produced by ARGextender on urban sewage samples and the ResFinder database (see Figure 3 in the main text). Looking at the computational time and memory usage in Figure B8, it is quite clear that ARGextender is a quicker and less memory-heavy alternative to SPAdes.

For the 29 sequencing runs, we evaluated the time and memory requirements for ARGextender using sequences from PanRes. Here, it is even clearer that for those samples that did finish, it took about a day and was relatively cheap in terms of memory (Figure B9). It did take more time to use ARGexender on PanRes than just ResFinder, which is likely due to the larger amount of sequences included in PanRes.

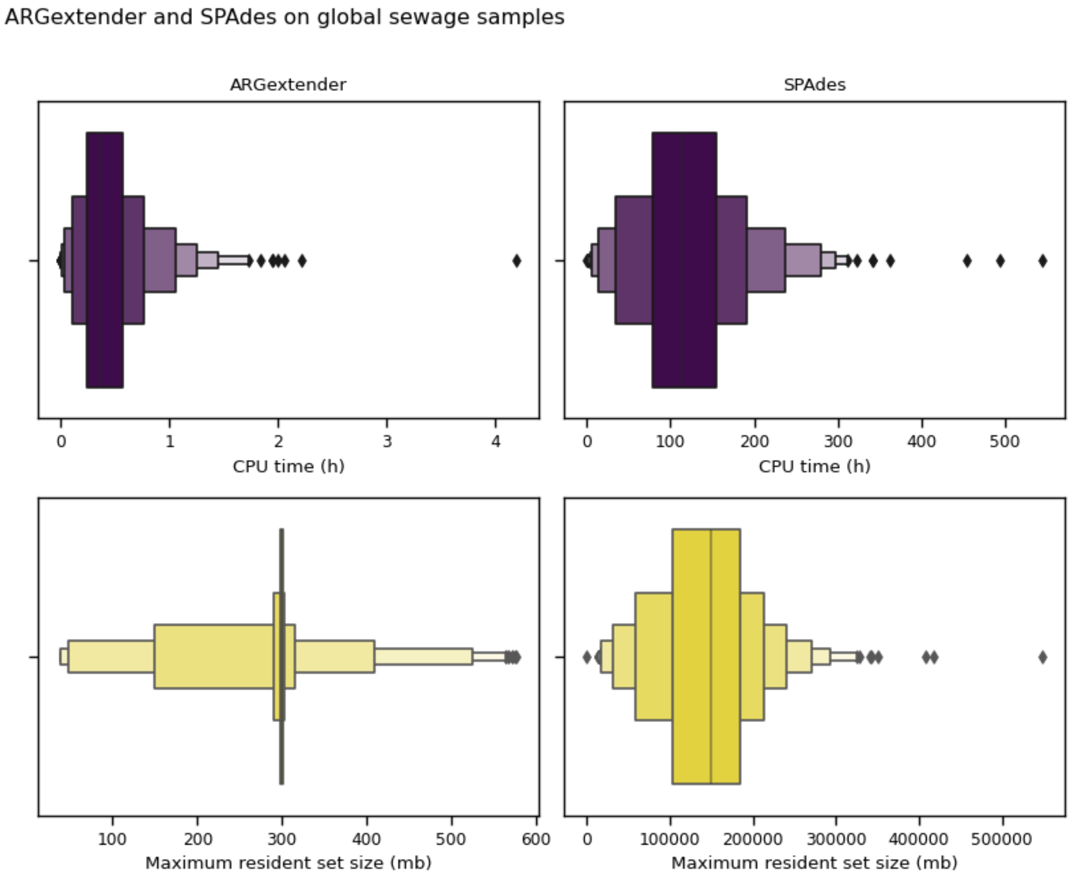

Figure B8. Time (hours) and memory (megabytes; mb) requirements for creating flanks around ARGs using ARGextender (left) or SPades (right). Note here that the flanks are created around ARGs from the ResFinder database.

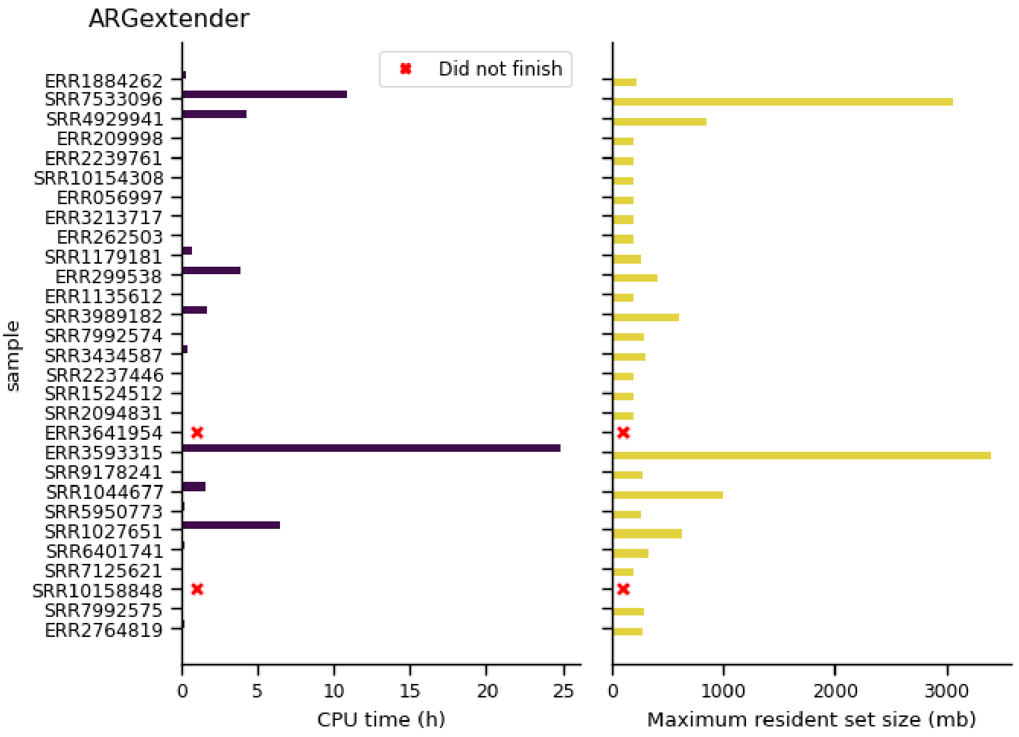

Figure B9. Time (hours) and memory (megabytes; mb) requirements for creating flanks around ARGs using ARGextender. Note here that the flanks are created around ARGs from the PanRes database. and that two of the runs did not finish within the 48-hour timeframe, marked with a red cross.

Total estimates for ARGprofiler per raw read datasets

Since ARGprofiler is capable of taking raw reads as input and then producing various outputs, we wanted to see how the total time and memory usage are given different sample sizes. Figure B10 shows these two usage statistics given the number of gigabases of raw reads, where it is quite clear that with an increased amount of gigabases of input, ARGprofiler will use more resources.

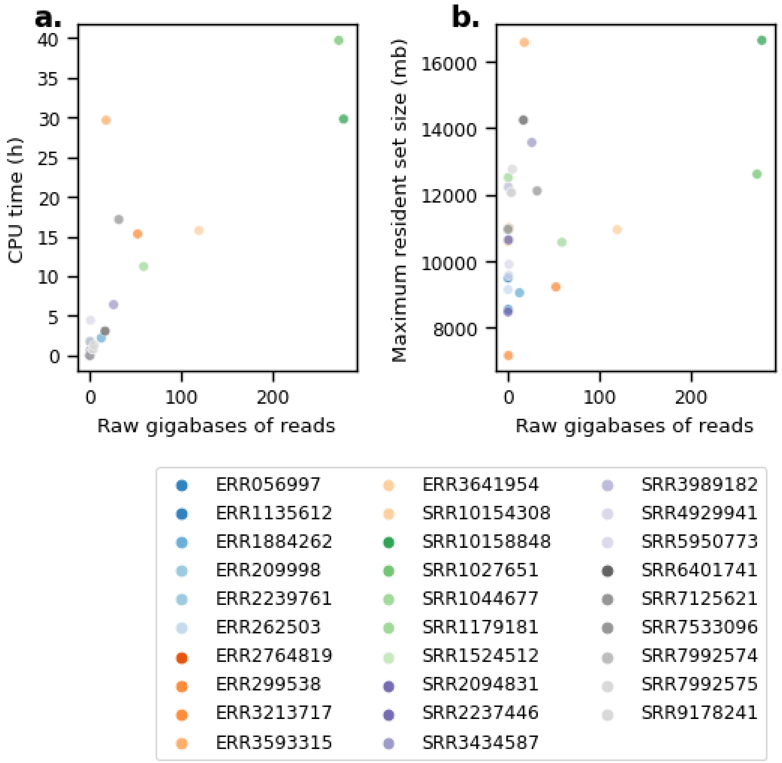

Figure B10. Total amount of a. time (hours) and memory (mb) ARGprofiler used per input of raw sequencing data.

References

Bortolaia V, Kaas RS, Ruppe E *et al.* ResFinder 4.0 for predictions of phenotypes from genotypes. *J Antimicrob Chemother* 2020;**75**:3491–500.

Bushnell B. BBMap. 2014.

Chen S. Ultrafast one-pass FASTQ data preprocessing, quality control, and deduplication using fastp. *iMeta* 2023;**2**:e107.

Clausen PTLC. Scaling neighbor joining to one million taxa with dynamic and heuristic neighbor joining. *Bioinformatics* 2023;**39**:btac774.

Martiny H-M, Munk P, Brinch C *et al.* A curated data resource of 214K metagenomes for characterization of the global antimicrobial resistome. Bollenbach T (ed.). *PLOS Biol* 2022;**20**:e3001792.

Ondov BD, Treangen TJ, Melsted P *et al.* Mash: fast genome and metagenome distance estimation using MinHash. *Genome Biol* 2016;**17**:132.

Quast C, Pruesse E, Yilmaz P *et al.* The SILVA ribosomal RNA gene database project:

Improved data processing and web-based tools. *Nucleic Acids Res* 2013;**41**:590–6.
Zankari E, Hasman H, Cosentino S *et al.* Identification of acquired antimicrobial resistance genes. *J Antimicrob Chemother* 2012;**67**:2640–4.
